# Distribution of glycated haemoglobin and its determinants in Korean youth and young adults: a nationwide population-based study

**DOI:** 10.1038/s41598-018-20274-8

**Published:** 2018-01-31

**Authors:** Ji-Young Seo, Seung-sik Hwang, Jae Hyun Kim, Young Ah Lee, Seong Yong Lee, Choong Ho Shin, Sei Won Yang

**Affiliations:** 1 Department of Pediatrics, Nowon Eulji Medical Center, Eulji University, Seoul, 01830 Korea; 2 Department of Public Health Science, Graduate School of Public Health, Seoul National University, Seoul, 08826 Korea; 3 Department of Pediatrics, Seoul National University Bundang Hospital, Seongnam, Gyeonggi-do 13620 Korea; 4 Department of Pediatrics, Seoul National University Children’s Hospital, Seoul National University College of Medicine, Seoul, 03080 Korea; 5 Department of Pediatrics, Seoul Metropolitan Government-Seoul National University Boramae Medical Center, Seoul, 07061 Korea

## Abstract

The present study aimed to describe the distribution of and to investigate the factors associated with glycated haemoglobin (HbA1c) values in Korean youth (10–19 years old) and young adults (20–29 years old). Data from the Korea Health and Nutrition Examination Survey (2011–2015) were used. A total of 6,418 participants (male 3,140 [53.2%]) aged 10–29 years were included in the analysis. Percentiles of HbA1c were calculated and HbA1c values were compared according to age, sex, and associated factors. The mean HbA1c values (% [mmol/mol]) were 5.42 ± 0.01 (35.7 ± 0.1) for youths and 5.32 ± 0.01 (34.7 ± 0.1) for young adults (*P* < 0.001). Male participants showed significantly higher HbA1c level than females (*P* < 0.001). When age was grouped into 5-year intervals, HbA1c was the highest in those aged 10–14 years and the lowest in those aged 20–24 years. After controlling for confounding variables, the HbA1c values of youths and male participants were significantly higher than those of young adults and female participants. The present study provides nationally representative data on the distribution of HbA1c values in Korean youth and young adults. There were significant differences in the level of HbA1c according to age and sex.

## Introduction

Glycated haemoglobin (HbA1c) is used as a tool to diagnose diabetes mellitus (DM) and is the best indicator of glycaemic control in patients with DM^1^. In addition, HbA1c is a useful predictor of cardiovascular disease and metabolic syndrome. Its normal range in adult populations is up to 5.7% (39 mmol/mol)^2–4^. HbA1c levels in adults show age-, sex-, and ethnicity-related differences, implying the need to establish reference values according to relevant factors^5–7^. Only a few population-based studies on HbA1c reference values have been performed to date^8,9^; these have demonstrated differences in the distribution of HbA1c values based on age, sex, and ethnicity. However, there has been no population-based study on HbA1c reference values in Korean youths and young adults; the previously published studies contain no data on the distribution of HbA1c values in the Asian paediatric population.

Considering the rapidly increasing prevalence of obesity and type 2 DM in children and adolescents, and the increasing use of HbA1c as a marker of glucose metabolism, it is important to establish normative reference values for the Korean population. Normative studies of children and adolescents, mostly conducted in the USA, showed differences in the distribution of HbA1c values based on age; values tended to be higher during adolescence^5,8,9^. The aims of the present study were to describe the normative distribution of HbA1c values and to investigate the factors associated with HbA1c values in Korean youths and young adults, using nationally representative data.

## Results

### Clinical characteristics of participants

The data from the Korea National Health and Nutrition Examination Survey (KNHANES) (2011–2015) were divided into two age groups and analysed accordingly: 3,629 subjects (45.5%) were in the 10–19-year-old age group (youth group) while 2,789 subjects (54.5%) were in the 20–29-year-old age group (young adult group). There was no difference in the proportion of male subjects between the two groups (52.9% and 53.6%, respectively). In the young adult group, the proportion of obesity was 23.1%, which were higher than the rates in the youth group. The fasting glucose level, HbA1c value, and the proportion of subjects with a carbohydrate intake over the recommended daily intake were lower in the young adult group than in the youth group (Table 1).

**Table 1:** Clinical characteristics of study participants.

Variable	Youths (10–19 y) n = 3629 (45.5%) Estimated population = 5,168,911	Young adults (20–29 y) n = 2789 (54.5%) Estimated population = 6,182,733	*P*-value
Male (%)	1926 (52.9%)	1214 (53.6%)	0.622
Obesity (%)	468 (13.8%)	599 (23.1%)	<0.001
Income, upper (%)	2290 (59.6%)	1872 (66.0%)	<0.001
Parental education, high (%)	1612 (45.2%)	608 (28.6%)	<0.001
Parental history of diabetes (%)	178 (5.3%)	362 (12.6%)	<0.001
Fasting glucose (mmol/L)	4.99 ± 0.01	4.90 ± 0.01	<0.001
HbA1c, % (mmol/mol)	5.42 ± 0.01 (35.7 ± 0.1)	5.32 ± 0.01 (34.7 ± 0.1)	<0.001
Haemoglobin (g/dL)	14.15 ± 0.03	14.54 ± 0.04	<0.001
Physical activity^a^ (%)	473 (16.6%)	396 (16.3%)	0.778
Carbohydrate intake^b^ (%)	1251 (38.2%)	740 (31.0%)	<0.001

Data are expressed as the weighted mean ± standard error or weighted percent.

^a^Physical activity: moderate-to-vigorous physical activity ≥ 60 min/day.

^b^Carbohydrate intake: carbohydrate consumption ≥ 65% of daily requirement intake for Koreans.

### Distribution of glycated haemoglobin values

#### Reference values for HbA1c according to age and sex

**Figure 1:**
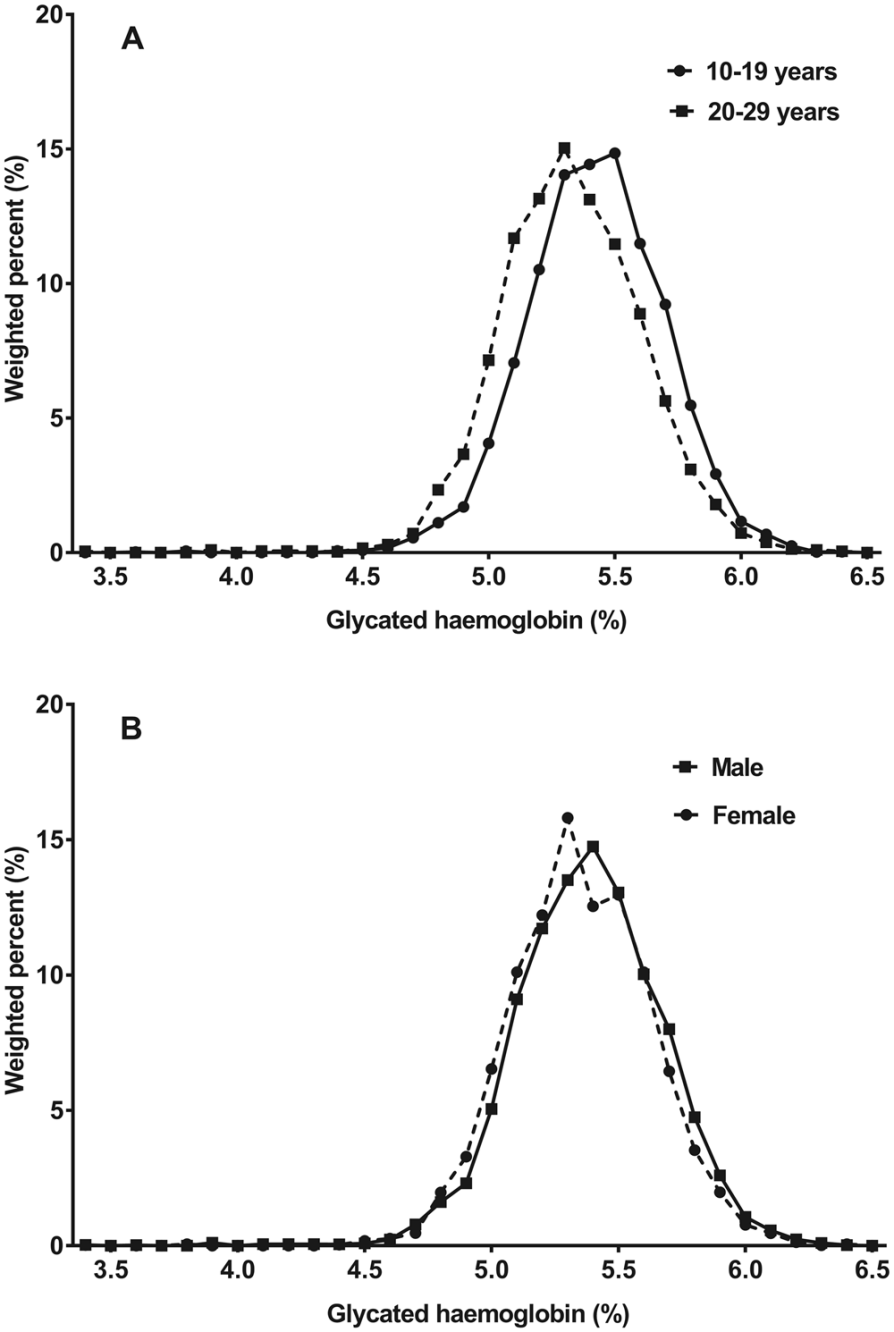
Distribution of glycated haemoglobin (HbA1c) by age group (**A**) and sex (**B**).

The distribution of HbA1c according to age group and sex are presented in Fig. 1. HbA1c was slightly higher among boys (Fig. 1A) and among those aged 10–19 years (Fig. 1B).

Table 2 shows the mean, standard error, and percentile values for serum HbA1c values according to age, sex, obesity, abdominal obesity, income, history of parental diabetes, physical activity level, and carbohydrate consumption. The overall mean and 90^th^ percentile values of HbA1c were 5.37% (35.2 mmol/mol) and 5.7% (39 mmol/mol), respectively. With respect to HbA1c value based on sex, male subjects had significantly higher values than female subjects, although the 90^th^ percentile value was same for both sexes (5.7%, 39 mmol/mol). The HbA1c value was significantly higher in those with obesity than in those without, while the 90^th^ percentile values for those with and without parental history of DM were 5.8% (40 mmol/mol) and 5.7% (39 mmol/mol), respectively. There was no difference in mean HbA1c value based on income. Individuals with a parental history of diabetes had higher HbA1c values, with a 90^th^ percentile value of 5.8% (40 mmol/mol).

**Table 2:** Distribution of glycated haemoglobin (HbA1c) values by age, sex, and associated factors.

		Mean	SE	Percentile of HbA1c									*P*-value
				1	5	10	25	50	75	90	95	99	
Total		5.37 (35.2)	0.01 (0.1)	4.7 (28)	4.9 (30)	5.0 (31)	5.2 (33)	5.4 (36)	5.6 (39)	5.7 (40)	5.9 (41)	6.0 (42)	
Age (y)	10–19	5.42 (35.7)	0.01 (0.1)	4.8 (29)	5.0 (31)	5.1 (32)	5.2 (33)	5.4 (36)	5.6 (38)	5.8 (40)	5.9 (41)	6.0 (42)	<0.001
	20–29	5.32 (34.7)	0.01 (0.1)	4.7 (28)	4.9 (30)	5.0 (31)	5.1 (32)	5.3 (34)	5.5 (37)	5.7 (39)	5.8 (40)	6.0 (42)	
Sex	Male	5.38 (35.3)	0.01 (0.1)	4.7 (28)	4.9 (30)	5.0 (31)	5.2 (33)	5.4 (36)	5.6 (38)	5.7 (39)	5.9 (41)	6.0 (42)	<0.001
	Female	5.35 (35.0)	0.01 (0.1)	4.7 (28)	4.9 (30)	5.0 (31)	5.2 (33)	5.3 (34)	5.5 (37)	5.7 (39)	5.9 (41)	6.0 (42)	
Age and sex	Male (10–19 y)	5.43 (35.9)	0.01 (0.1)	4.7 (28)	5.0 (31)	5.1 (32)	5.3 (34)	5.4 (36)	5.6 (38)	5.8 (40)	5.9 (41)	6.1 (43)	<0.001
	Female (10–19 y)	5.41 (35.6)	0.01 (0.1)	4.8 (29)	5.0 (31)	5.1 (32)	5.2 (33)	5.4 (36)	5.6 (38)	5.7 (39)	5.8 (40)	6.0 (42)	
	Male (20–29 y)	5.34 (34.9)	0.01 (0.1)	4.6 (27)	4.9 (30)	5.0 (31)	5.2 (33)	5.3 (34)	5.5 (37)	5.7 (39)	5.8 (40)	6.0 (42)	<0.001
	Female (20–29 y)	5.31 (34.5)	0.01 (0.1)	4.7 (28)	4.9 (30)	5.0 (31)	5.1 (32)	5.3 (44)	5.5 (37)	5.6 (38)	5.8 (40)	6.0 (42)	
Obesity^a^	No	5.35 (35.0)	0.01 (0.1)	4.7 (28)	4.9 (30)	5.0 (31)	5.2 (33)	5.4 (36)	5.5 (37)	5.7 (39)	5.8 (40)	6.0 (42)	<0.001
	Yes	5.43 (35.8)	0.02 (0.1)	4.7 (28)	5.0 (31)	5.1 (32)	5.2 (33)	5.4 (36)	5.6 (38)	5.8 (40)	5.9 (41)	6.1 (43)	
Income	Lower	5.37 (35.2)	0.01 (0.1)	4.8 (29)	4.9 (30)	5.0 (31)	5.2 (33)	5.4 (36)	5.6 (38)	5.7 (39)	5.8 (40)	6.0 (42)	0.899
	Upper	5.36 (35.2)	0.01 (0.1)	4.7 (28)	4.9 (30)	5.0 (31)	5.2 (33)	5.4 (36)	5.6 (38)	5.7 (39)	5.8 (40)	6.0 (42)	
Parental education	Low	5.37 (35.1)	0.01 (0.1)	4.7 (28)	4.9 (30)	5.0 (31)	5.2 (33)	5.4 (36)	5.6 (38)	5.7 (39)	5.8 (40)	6.0 (42)	0.036
	High	5.39 (35.4)	0.01 (0.1)	4.8 (29)	5.0 (31)	5.1 (32)	5.2 (33)	5.4 (36)	5.6 (38)	5.7 (39)	5.8 (40)	6.0 (42)	
History of parental DM	No	5.37 (35.1)	0.01 (0.1)	4.7 (28)	4.9 (30)	5.0 (31)	5.2 (33)	5.4 (36)	5.6 (38)	5.7 (39)	5.8 (40)	6.0 (42)	0.027
	Yes	5.40 (35.5)	0.01 (0.2)	4.7 (28)	5.0 (31)	5.1 (32)	5.2 (33)	5.4 (36)	5.6 (38)	5.8 (40)	5.9 (41)	6.1 (43)	

HbA1c was described as NGSP, % (IFCC, mmol/mol).

^a^Obesity: body mass index ≥95^th^ percentile for age and sex or body mass index. ≥25 kg/m^2^.

SE, standard error; DM, diabetes mellitus.

**Figure 2:**
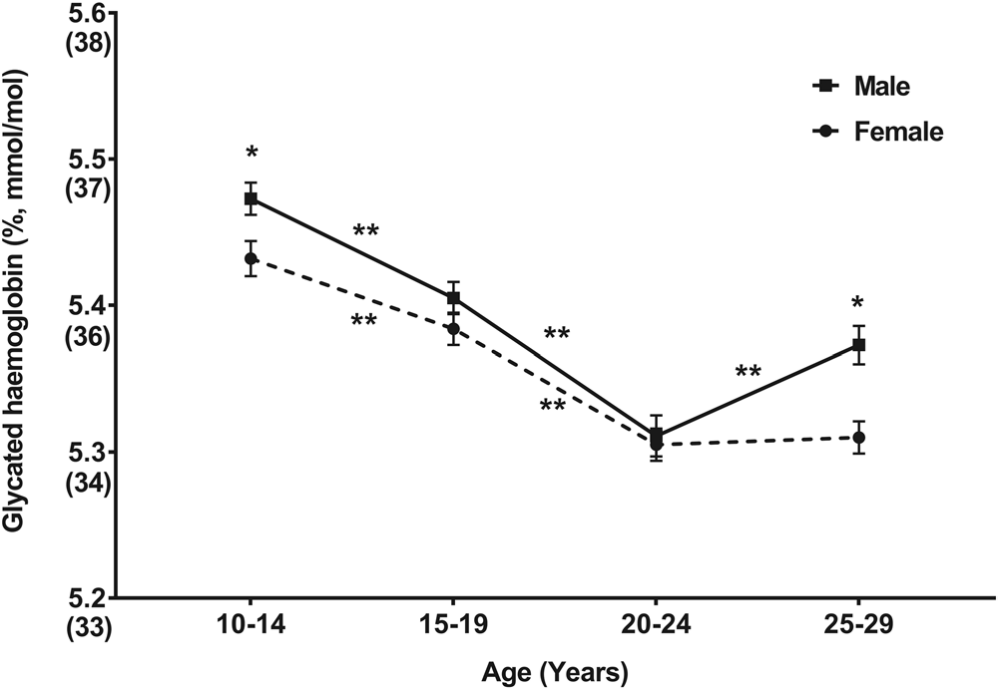
Difference in Glycated Haemoglobin (Weighted Mean and Standard Error) According to Age Group and Sex. (*P < 0.05 between sex, **P < 0.05 between adjacent age groups).

The results of the analysis stratified into four age subgroups showed that HbA1c values were significantly highest in the 10–14-year-old and lowest in the 20–24-year-old subgroups (Fig. 2). HbA1c level of males was significantly higher in the 10–14-year-old and 25–59-year-old groups.

#### Polynomial regression analysis

**Figure 3:**
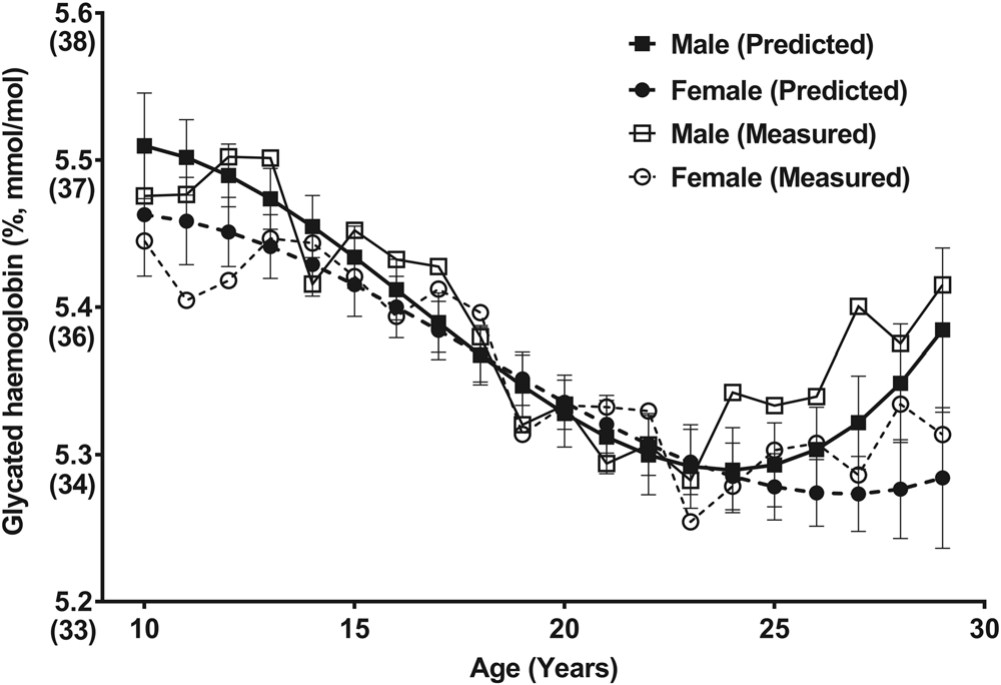
Predicted glycated haemoglobin with 95% confidence intervals by age and sex based on a polynomial model (adjusted R^2^ = 0.056, *P* < 0.001).

The best-fitted model selected after polynomial regression analysis had the adjusted R-square of 0.056, which was statistically significant (*P* < 0.001) (Fig. 3 and Table 3). The structure of polynomial regression that we assumed (Table 3) is as follows:

$$\begin{array}{ccc}{\rm{H}}{\rm{b}}{\rm{A}}1{\rm{c}}({\rm{ \% }}) & = & 4.9500+0.0879\times {\rm{a}}{\rm{g}}{\rm{e}}-0.0066\times {{\rm{a}}{\rm{g}}{\rm{e}}}^{2}\\  &  & +\,0.0001\times {{\rm{a}}{\rm{g}}{\rm{e}}}^{3}+0.0821\times {\rm{s}}{\rm{e}}{\rm{x}}\\  &  & -\,0.0347\times {\rm{s}}{\rm{e}}{\rm{x}}\times {\rm{a}}{\rm{g}}{\rm{e}}+0.0028\times {\rm{s}}{\rm{e}}{\rm{x}}\times {{\rm{a}}{\rm{g}}{\rm{e}}}^{2}\\  &  & -\,0.0001\times {\rm{s}}{\rm{e}}{\rm{x}}\times {{\rm{a}}{\rm{g}}{\rm{e}}}^{3}+0.0099\times {\rm{B}}{\rm{M}}{\rm{I}}+0.0738\\  &  & \times \,({\rm{f}}{\rm{a}}{\rm{m}}{\rm{i}}{\rm{l}}{\rm{y}}\,{\rm{h}}{\rm{i}}{\rm{s}}{\rm{t}}{\rm{o}}{\rm{r}}{\rm{y}}\,{\rm{o}}{\rm{f}}\,{\rm{D}}{\rm{M}})-0.0019\times ({\rm{h}}{\rm{o}}{\rm{u}}{\rm{s}}{\rm{e}}\,{\rm{i}}{\rm{n}}{\rm{c}}{\rm{o}}{\rm{m}}{\rm{e}})\\  &  & +\,0.0058\times ({\rm{p}}{\rm{a}}{\rm{r}}{\rm{e}}{\rm{n}}{\rm{t}}{\rm{a}}{\rm{l}}\,{\rm{e}}{\rm{d}}{\rm{u}}{\rm{c}}{\rm{a}}{\rm{t}}{\rm{i}}{\rm{o}}{\rm{n}}).\end{array}$$

**Table 3:** Polynomial regression analysis predicting level of glycated haemoglobin (%) (adjusted R^2^ = 0.056, *P* < 0.001).

Variable		Adjusted coefficient (95% confidence interval)	*P* value
Sex	Female	0.0821 (−0.6580 to 0.8222)	0.828
	Male	Ref	
Sex × Age	Female	−0.0347 (−0.1625 to −0.0930)	0.594
	Male	Ref	
Sex × Age^2^	Female	0.0028 (−0.0042 to 0.0098)	0.430
	Male	Ref	
Sex × Age^3^	Female	−0.0001 (−0.0002 to 0.0001)	0.308
	Male	Ref	
Age		0.0879 (0.0315 to 0.1594)	0.056
Age^2^		−0.0066 (−0.0117 to −0.0017)	0.009
Age^3^		0.0001 (0.0000 to 0.0002)	0.002
Body mass index		0.0099 (0.0072 to 0.0127)	<0.001
Parental history of diabetes	Yes	0.0738 (0.0401 to 0.1075)	<0.001
	No	Ref	
House income	Upper	−0.0019 (−0.0248 to 0.0211)	0.873
	Lower	Ref	
Parental Education	High	0.0058 (−0.0160 to 0.0275)	0.603
	Low	Ref	

CI, confidence interval; Ref, reference.

Age^2^, age^3^, BMI and parental history of DM were significantly associated with the level of HbA1c.

## Discussion

In the present study, we determined the reference values of HbA1c in Korean youths and young adults aged 10–29 years, using a nationally representative survey sample. The overall mean HbA1c value was 5.37% (35.2 mmol/mol). The mean HbA1c value was 5.42% (35.7 mmol/mol) in youths (10–19 years old) and 5.32% (34.7 mmol/mol) in young adults (20–29 years). In the sex-stratified analysis, the mean HbA1c values in male and female subjects were 5.38% (35.3 mmol/mol) and 5.35% (35.0 mmol/mol), respectively. These mean values are higher than those reported for children and adolescents of other race groups and ethnicities^8,10^. The mean HbA1c values reported by Saaddine *et al*.^8^ using the Third National Health and Nutrition Examination Survey (including 7968 subjects aged 5–24 years) were 4.93 ± 0.04% in non-Hispanic white, 5.05 ± 0.02% in Mexican-American, and 5.17 ± 0.02% in non-Hispanic black subjects. In a subsequent analysis^9^, these ethnic and racial differences persisted, even after controlling for age, sex, BMI, maternal BMI, and poverty-income ratio.

HbA1c values have been demonstrated to show racial differences, where African-American and Hispanic populations consistently show higher values than Caucasian populations^11–13^, regardless of fasting glucose and post-prandial glucose concentrations^14^. The reasons for such ethnic and racial differences in HbA1c levels are not yet fully understood. Among Asians, a high carbohydrate intake may be one of the causes, but the regression analysis in the present study did not find such a correlation. Other suspected causes include difference in the glycation of haemoglobin^15–17^, variation in red cell turnover^18^, and differences in the intraerythrocyte and extraerythrocyte environment^19^. However, such comparisons could not be made due to lack of studies on the HbA1c values of youth and young adults in other Asian regions.

Because Fig. 2 is suggestive of a third-degree polynomial fit, we used a polynomial regression model with cubic term for ‘age’ to predict the value of HbA1c. We also considered only a linear and quadratic term for age, but the cubic model was chosen because it had the larger adjusted R-square value (adjusted R^2^ = 0.056) and the model was statistically significant (*P* < 0.001). The cubic term provided a better fit to the data than the simple linear and quadratic term for age (data not shown). The term of interest in the polynomial regression model is the interaction term, which indicates that the HbA1c level varies by age, depending on the sex of the individual. However, the HbA1c level was insufficiently explained considering all variables aforementioned (adjusted R^2^ = 0.056).

In the present study, HbA1c was higher in the 10–19-year-old age group than in the 20–29-year-old age group (Fig. 2), with the group aged 10–14 years showing the highest values; these findings are compatible with those of other studies^8^. Carbohydrate intake was also higher in the 10–19-year-old age group, but it was interesting that the younger age groups had higher HbA1c values and fasting glucose concentrations despite the higher obesity ratios and a higher proportion of subjects with a parental history of diabetes in the 20–29-year-old age group. This may be linked to a decrease in insulin sensitivity during adolescence^20^. However, insulin sensitivity and resistance could not be compared between two groups in the present study due to the limited data on insulin.

HbA1c level was higher among male subjects, which is consistent with the findings of other studies (Fig. 1)^8,9^. However, the reason for this remains unclear. The level of HbA1c was negatively correlated with haemoglobin concentration, where haemoglobin concentrations were lower in those aged 20–29 years than in those aged 10–19 years, and in female than in male subjects. Despite such findings, HbA1c values were actually higher in youths (10–19 years) and in male subjects. Thus, it can be speculated that factors other than haemoglobin concentration, such as insulin resistance, may have a greater influence on the HbA1c level. As age increased, HbA1c tended to decrease, reaching the minimum value in the 20–24-year-old age group. The value increased again in male subjects aged 25–29 years, but remained stable in female subjects. In all age groups except 20–24 years, male subjects had higher HbA1c values than female subjects.

HbA1c values were higher in those with a history of parental diabetes; given that these subjects have a genetic predisposition to developing DM, HbA1c levels may be affected by such a factor. In the present study, household income level and parental educational level, representing socio-economic status, showed no statistically significant correlations with HbA1c values.

HbA1c is used as a diagnostic criterion for diabetes and prediabetes; the cut-off values generally applied for prediabetes and diabetes are 5.7% (39 mmol/mol) and 6.5% (48 mmol/mol), respectively, but in the present study, 5.7% (39 mmol/mol) corresponded approximately to the 90^th^ percentile value, while 6.5% (48 mmol/mol) corresponded to over the 99^th^ percentile value. According to data from the USA^8^, the 90^th^ and 95^th^ percentile values for those aged 5–24 years are 5.39% and 5.52%, respectively, which are lower than the values for Koreans obtained in the present study. The cut-off value for prediabetes in the present study, corresponding to the 90^th^ percentile value, bears sufficient statistical significance, and since the cut-off value for diabetes well exceeded the 99^th^ percentile value, applying an HbA1c value of 6.5% as a diagnostic criterion for DM in youths and young adults in Korea would yield higher positive predictive values and specificity than in other ethnicities.

In the present study, a higher proportion of subjects with than without an HbA1c result had obesity. However, prevalence of obesity in the subjects whose data were included in the present analysis was similar to that of the general Korean population, and thus, the test results may be considered reliable.

The present study had the limitations of being a cross-sectional study and lacking data for younger ages (<10 years). Longitudinal data would be more helpful for the understanding of the change in HbA1c according to the change in individual risk factors. However, it is the first study to use national data to determine the distribution of HbA1c values for the Korean population. It should be noted that the findings indicate much higher mean HbA1c values in Korean youths and young adults than in their peers in Western countries.

In conclusion, normative values of HbA1c in Korean youth and young adults were presented. An HbA1c value of 5.7% (39 mmol/mol) corresponds approximately to the 90^th^ percentile, while a value of 6.5% (48 mmol/mol) corresponds to over the 99^th^ percentile. The reference values for HbA1c are higher in male than female subjects and are markedly higher in this study population than values reported in similar age groups in Western countries. Therefore, age, sex and ethnic background should be considered for interpretation of HbA1c level.

## Methods

### Study population

Data from the KNHANES for the period 2011–2015 were used for the analysis^21^. KNHANES is an ongoing series of population-based cross-sectional surveys assessing the health and nutrition of Koreans. A multi-stage clustered probability sampling design was used to obtain a nationally representative sample of non-institutionalized Korean civilians. The KNHANES comprises health interviews, health examinations, and nutritional surveys. The target population of the KNHANES comprises noninstitutionalised Korean citizens residing in Korea. The sampling plan follows a multi-stage clustered probability design. Primary sample units were drawn from whole country. Detailed descriptions of the study design and data collection methods have been published elsewhere^22^. Since 2011, HbA1c measurement has been included in the health examination of participants aged ≥10 years. The response rates were 80.8% for the period 2011–2012 and 78.3% for the period 2013–2015.

Of the 39,524 participants of KNHANES 2011–2015, 7,978 (male 3,888 [48.7%]) subjects aged 10–29 years were selected as potential candidates. Of these, 1,560 (19.6%) subjects were excluded for the following reasons: no record of HbA1c being measured (*n* = 1,454), having a diagnosis of DM (*n* = 85), HbA1c value ≥ 6.5% (48 mmol/mol) (*n* = 29), and pregnant at the time of examination (*n* = 40). Thus, a final sample of 6,418 subjects (male, *n* = 3,140; female, *n* = 3,278) was included in the analysis.

There was no significant difference between subjects with and without an HbA1c result in terms of the proportion with obesity, parental history of DM, level of physical activity, amount of carbohydrate intake, and haemoglobin concentration. Among those with HbA1c results, a greater proportion of subjects were male (53.2% vs. 48.4%, *P* = 0.015) and had a high household income (63.1% vs. 56.7%, *P* = 0.003). HbA1c measurement was performed in 84.3% and 90.7% of participants aged 10–19 years and 20–29 years, respectively (*P* < 0.001)

The KNHANES was approved by the Institutional Review Board of the Korea Centers for Disease Control and Prevention. All participants provided informed consent before data collection. The present study protocol was approved by the Institutional Review Board of Seoul National University Bundang Hospital. All methods were performed in accordance with the Declaration of Helsinki.

### Laboratory measurements and lifestyle evaluation

Blood samples were obtained by trained medical personnel after a fasting period of at least 8 h. The samples were analysed within 24 h of collection in a central laboratory. HbA1c (%) was measured using high performance liquid chromatography (HLC-723G7; Tosoh, Tokyo, Japan), which is the method certified by the National Glycohemoglobin Standardization Program (NGSP)^23^. HbA1c (%) values were converted into SI unit recommended by the International Federation of Clinical Chemistry (IFCC) using the master equation: IFCC (mmol/mol) = 10.93 × NGSP (%) − 23.52. Haemoglobinopathies were not considered in the analysis of HbA1c because of the extremely low prevalence of variant haemoglobin in the Korean population^24^. Plasma glucose concentrations were measured by the hexokinase method using a Hitachi Automatic Analyzer 7600 (Hitachi, Tokyo, Japan). Haemoglobin was measured by the sodium lauryl sulphate detection method, using an XE-2100D™ haematology analyzer (Sysmex®, Kobe, Japan).

Obesity was defined as a body mass index (BMI) ≥95^th^ percentile for age and sex or ≥25 kg/m^2^. Individual lifestyle factors were assessed using the level of physical activity and amount of carbohydrates consumed. In terms of physical activity, subjects were categorized into two groups: those who engaged in ≥60 min of moderate-to-vigorous physical activity per day and those who did not. In terms of carbohydrate intake, subjects were divided into two groups: those who exceeded the upper limit of the recommended daily intake for Koreans (>65%) and those who did not^25^. As an indicator of socioeconomic status, household income and parental education level were used. Household income was categorized into an upper and lower group according to the two upper and lower quartiles, respectively. To assess parental education level, the highest education level of either one of the parents/caregivers was used, categorized as university graduation or more and up to high school. A parental history of DM was used as a marker of genetic factors^8–10,26^.

### Statistical analysis

Stata Statistical Software, Release 14.2 (StataCorp LP, College Station, Texas, USA) and GraphPad Prism version 7.03 for Windows (GraphPad Software, La Jolla, California, USA) were used for the statistical analysis. The *svy* command was used for the analysis, taking sample weights into account; this approach was appropriate for the design of the KNHANES. The sample weights were constructed for sample participants to represent the Korean population by accounting for the complex survey design, survey non-response and post-stratification. The weights based on the inverse of selection probabilities and inverse of response rates were modified by adjusting them to the sex- and age-specific Korean populations (post-stratification). Data are presented as weighted means ± standard errors (SE) for continuous variables or as the number of cases with a weighted percentage for categorical variables. Student’s *t*-test was used to compare continuous variables and the chi-square test was used to compare categorical variables. Distribution curves were generated according to age group and sex. Mean HbA1c values were compared between age groups (divided into 5-year intervals) and by sex.

Polynomial regression analysis was performed to predict HbA1c levels. The independent variables included age, sex, BMI, parental history of DM, the level of household income and parental education level. Because the mean HbA1c value for age showed a third-degree polynomial fit, the best fitted model estimated using a cubic fit was selected, using a cubic term for age. *P*-values < 0.05 were considered statistically significant.

## References

[1] Nathan DM, Singer DE, Hurxthal K, Goodson JD (1984). The clinical information value of the glycosylated hemoglobin assay. N. Engl. J. Med..

[2] The International Expert Committee (2009). International Expert Committee Report on the Role of the A1C Assay in the Diagnosis of Diabetes. Diabetes Care..

[3] American Diabetes Association (2010). Standards of Medical Care in Diabetes-2010. Diabetes Care..

[4] Ko SH (2011). 2011 Clinical Practice Guidelines for Type 2 Diabetes in Korea. Diabetes Metab. J..

[5] Avilés-Santa ML (2016). Differences in Hemoglobin A1c Between Hispanics/Latinos and Non-Hispanic Whites: An Analysis of the Hispanic Community Health Study/Study of Latinos and the 2007–2012 National Health and Nutrition Examination Survey. Diabetes Care..

[6] Christensen DL (2010). Moving to an A1C-based diagnosis of diabetes has a different impact on prevalence in different ethnic groups. Diabetes Care..

[7] Du T, Yuan G, Zhou X, Sun X (2016). Sex differences in the effect of HbA1c-defined diabetes on a wide range of cardiovascular disease risk factors. Ann. Med..

[8] Saaddine JB (2002). Distribution of HbA(1c) Levels for Children and Young Adults in the U.S.: Third National Health and Nutrition Examination Survey. Diabetes Care..

[9] Eldeirawi K, Lipton RB (2003). Predictors of hemoglobin A1c in a national sample of nondiabetic children: the Third National Health and Nutrition Examination Survey, 1988–1994. Am. J. Epidemiol..

[10] Carson AP (2016). Do glycemic marker levels vary by race? Differing results from a cross-sectional analysis of individuals with and without diagnosed diabetes. BMJ Open Diabetes Res. Care..

[11] Kirk JK (2006). Disparities in HbA_1C_ Levels Between African-American and Non-Hispanic White Adults With Diabetes: A meta-analysis. Diabetes Care..

[12] Herman WH (2007). Differences in A1C by Race and Ethnicity Among Patients With Impaired Glucose Tolerance in the Diabetes Prevention Program. Diabetes Care..

[13] Ziemer DC (2010). Glucose-independent, black-white differences in hemoglobin A1c levels: a cross-sectional analysis of 2 studies. Ann. Intern. Med..

[14] Bergenstal RM (2017). Racial Differences in the Relationship of Glucose Concentrations and Hemoglobin A1c Levels. Ann. Intern. Med..

[15] Snieder H (2001). HbA(1c) levels are genetically determined even in type 1 diabetes: evidence from healthy and diabetic twins. Diabetes..

[16] Cohen RM (2006). Evidence for independent heritability of the glycation gap (glycosylation gap) fraction of HbA1c in nondiabetic twins. Diabetes Care..

[17] Cohen RM, Franco RS, Khera PK (2008). Red cell life span heterogeneity in hematologically normal people is sufficient to alter HbA1c. Blood..

[18] Khera PK (2008). Evidence for Interindividual Heterogeneity in the Glucose Gradient Across the Human Red Blood Cell Membrane and Its Relationship to Hemoglobin Glycation. Diabetes..

[19] Amiel SA, Sherwin RS, Simonson DC, Lauritano AA, Tamborlane WV (1986). Impaired insulin action in puberty. A contributing factor to poor glycemic control in adolescents with diabetes. N. Engl. J. Med..

[20] Korea Centers for Disease Control and Prevention. The 6th Korea National Health and Nutrition Examination Survey Source Data. https://knhanes.cdc.go.kr/knhanes/sub03/sub03_02_02.do (2017).

[21] Kweon S (2014). Data resource profile: the Korea National Health and Nutrition Examination Survey (KNHANES). Int. J. Epidemiol..

[22] Holmes EW (2008). Analytic bias among certified methods for the measurement of hemoglobin A1c: a cause for concern?. Am. J. Clin. Pathol..

[23] Park ES (2013). Hereditary hemolytic anemia in Korea from 2007 to 2011: A study by the Korean Hereditary Hemolytic Anemia Working Party of the Korean Society of Hematology. Blood Res..

[24] Ministry of Health and Welfare, The Korean Nutrition Society. Dietary Reference Intakes for Koreans 2015. Sejong, 2015.

[25] Pettitt DJ, Giammattei J, Wollitzer AO, Jovanovic L (2004). Glycohemoglobin (A1C) distribution in school children: results from a school-based screening program. Diabetes Res. Clin. Pract..

[26] Jansen H (2009). HbA(1c) levels in non-diabetic Dutch children aged 8-9 years: the PIAMA birth cohort study. Diabet. Med..

